# Animal-based radiation absorbed dose evaluation of holmium-166 labeled hydroxyapatite particulates in liver malignancies

**DOI:** 10.22038/aojnmb.2024.79679.1560

**Published:** 2025

**Authors:** Reza Bagheri, Hassan Ranjbar

**Affiliations:** 1 Radiation Applications Research School, Nuclear Science and Technology Research Institute, Tehran, Iran; 2 Nuclear Fuel Cycle Research School, Nuclear Science and Technology Research Institute, Tehran, Iran

**Keywords:** ^166^Ho-HA, Liver cancer therapy Absorbed dose, MIRD method, Wistar rat

## Abstract

**Objective(s):**

Liver malignancies are among the most prevalent causes of cancer-related deaths worldwide. Intra-arterial administration of particulates labeled with beta-emitting radionuclides is one of the non-surgical promising modalities for the treatment of liver cancer.

**Methods:**

In this work, the radiation absorbed dose of ^166^Ho-hydroxyapatite (^166^Ho-HA) radiopharmaceutical was estimated for adult men based on biodistribution data in normal Wistar rats. The MIRD dose calculation method and the Sparks and Aydogan methodology were applied.

**Results:**

The results show that more than 84% of the absorbed dose is localized in liver tissue (7.35 mGy MBq^-1^). Also, radiation absorbed doses of ^166^Ho-HA for red bone marrow, osteogenic cells, and spleen tissues were estimated to be about 0.18, 0.38, and 0.24 mGy MBq^-1^, respectively. The maximum administrated activity was obtained at 87.5 MBq kg^-1^ of body weight with an effective dose of 0.39 mSv MBq^-1^. The maximum tolerable dose (MTD) for liver tissue was 6.13 GBq (165.56 mCi).

**Conclusion:**

This study indicated that ^166^Ho-HA can provide an impressive dose for liver cancer malignancies with an insignificant dose to healthy tissues.

## Introduction

 Primary and secondary liver malignancies are among the most prevalent causes of cancer-related deaths worldwide (1, 2). Although surgical resection, systemic chemotherapy, as well as regional administration of chemotherapeutic drugs, and external radiotherapy, are the conventional treatment modalities for liver cancers, most of them have shown little improvement in patient survival, especially in metastatic liver cancer (3, 4). 

 Recently radionuclide therapy has been raised as a promising alternative treatment modality for liver cancer. Several radiolabeled microspheres or particulates such as ^186^Re/ ^188^Re glass microspheres, yttrium-90 labeled glass and resin microspheres, and holmium-166 labeled polylactic acid (PLA) microspheres have been used for liver cancer and liver metastases through the hepatic artery administration of desired radiopharmaceutical (3,5-9). Holmium-166 labeled hydroxyapatite particulates (^166^Ho-HA) can be considered an attractive radiopharmaceutical for intra-arterial therapy of liver cancers owing to the high energy beta particle accompanying gamma emission for simultaneous scintigraphic imaging (10).

 Holmium-166 with an appropriate half-life of 1.12 days, two relatively high beta energies (1.85 MeV [51%] and 1.77 MeV [48%]), long penetration range in soft tissue (8.7 mm) and gamma-ray for imaging studies (81 keV, 6.7%) is an excellent radionuclide for delivering of interested dose for liver malignancies (11-13). 

 Hydroxyapatite (HA) is a natural mineral constituent of bone matrix, so they are biocompatible and biodegradable. HA particles can be easily synthesized in the desired particle sizes (20–60 µm) and can be easily radiolabeled with lanthanide radioisotopes, resulting in highly stable radiolabeled compositions (10, 14, 15).

 This study aims to estimate the radiation absorbed dose of ^166^Ho-HA radiopharma-ceutical for organs of an adult man based on biodistribution data in Wistar-type rats (10). 

 Previously published studies in the literature have justified the usefulness of animal models for initial absorbed dose evaluations (16-18). 

 Wherever possible, the result will be compared with other published data from the literature.

## Methods

***Biodistribution studies of*** ^166^ ***Ho-HA in Wistar rats***

 Production and quality control of ^166^Ho-HA radiopharmaceutical has been fully described by Das et al. (10). In this research, only biodistribution data in Wistar rats from the article mentioned above is used for radiation absorbed dose evaluation of ^166^Ho-HA in man. 

 Briefly, the radiolabeled hydroxyapatite particles with carrier-added holmium-166 radionuclide were produced by irradiation of natural Ho2O3 (100% ^165^Ho) at a thermal neutron flux of 3×1013 n cm-2 s for a period of 7 d. HA particles within the 20–60 µm range were carried out by repetitive grinding and sieving, using ASTM standard sieves of suitable mesh size. Characterization of the particles was carried out by X-ray diffractometry. The size of the particles obtained was analyzed by using a LASER diffraction particle-size analyzer. 

 Labeling of HA particles with holmium-166 was achieved by suspending 5 mg of the HA particles in 0.7 mL of normal saline, followed by the addition of 0.2 mL of 0.5 M NaHCO_3_ buffer (pH 9) and 0.1 mL of ^166^HoCl_3_ solution (containing about 370 MBq of Ho-166). The radiolabeling yield of the ^166^Ho-labeled HA particles was determined by counting the activity associated with the HA particles and supernatant solution. 

 For in-vitro stability studies, the radiolabeled particles were stored in normal saline and freshly separated human serum at room temperature and 37 °C.

 Biodistribution of ^166^Ho-HA was studied in normal Wistar rats injected with 0.1 mL (~5 MBq of ^166^Ho) of suspension of the radiolabeled particles in normal saline through the hepatic artery of rat post-anesthetization of each animal. The animals were sacrificed by cardiac puncture post-anesthesia at 1, 3, 24, and 48 h post-injection. Three rats were used in each time interval. After drawing blood from the aorta, organs were weighed and counted in a flat-type NaI(Tl) scintillation counter. The tissues’ radioactivities were stated as a percentage of injected activity per organ (%IA/organ).

^166^ ***Ho-HA's biodistribution in humans***

 The following methods are used to adapt the biodistribution pattern of ^166^Ho-HA radiopharmaceutical between rats and humans; the Medical Internal Radiation Dosimetry (MIRD) dose calculation method (19) and the methodology for extrapolation of the percent of injected activity (%IA) in human organ from the percent of injected activity (%IA) in animal organ. 

 Regarding the considerable difficulties in extrapolating biokinetic data from animals to humans, the methodology outlined by Sparks and Aydogan is applied in this study to have a rough approximation of radiation absorbed dose in man from ^166^Ho-HA (20). Based on this method, in relative organ mass scaling, the percent of injected activity (%IA) in human organ is assumed to be equal to the ratio of the fraction of the total body mass of the organ in human to the fraction of the total body mass of the organ in animal multiplied by the percent of injected activity (%IA) in animal organ:

IA Human organ = %IA Animal organ ×$\frac{\frac{{\mathrm{Organ mass}}_{\mathrm{human}}}{{\mathrm{Body mass}}_{\mathrm{human}}}}{\frac{{\mathrm{Organ mass}}_{\mathrm{animal}}}{{\mathrm{Body mass}}_{\mathrm{animal}}}}$ (1)

 Because the ^166^Ho-HA radiopharmaceutical is directly injected into the liver organ through the hepatic artery, so %IA of humans for this organ was considered equal to the rat’s value.

 The activity of organs in any time interval after injection of A_0_ Bq of ^166^Ho-HA is calculated from the following equation and the time-activity curves are produced for each source organ according to this equation:

*A(t)* =$\frac{\mathrm{\%IA}(t)}{100}\times A_{0}e^{-\mathrm{\lambda t}}$ (2)

***Dosimetric calculations***

 The radiation absorbed dose of interested target organs was estimated using methods recommended by the Medical Internal Radiation Dose (MIRD) Committee of the Society of Nuclear Medicine (19). The calculations based on the methodology described below:

*D(* $r_{T}$ *) =* $\sum_{r_{S}}Ã(r_{S})S(r_{T}\leftarrow r_{S})$ (3)

 Where, D(r_T_) stated in (mGy) is the radiation absorbed dose to a target organ, r_T_, from source organs, r_S_. $Ã\left(r_{S}\right)$ is the accumulated activity in the source organ, r_S_, which is calculated following equation:

$Ã(r_{S})=\int_{0}^{\infty }A\left(r_{S},t\right)\mathrm{dt}$ (4)

 And, the S(r_T_ ← r_S_) expressed in [mGy/(MBq s^-1^)], is the specific absorbed fraction of energy for the target organ, r_T_, per unit accumulated activity in the source organ, r_S_. In this study, the S-values of adult men for holmium-166 radionuclide were taken from the OLINDA/EXM software (21).

 The accumulated activity (total number of disintegrations) of each source organ was calculated in two steps. In the first step, due to the availability of biodistribution data up to 48 h post-injection, the time-activity curve of each source organ was integrated up to 48 h (2 days), and the area under the curve was considered as the accumulated activity until 48 h. In the second step, one mono-exponential function was considered as the rest of the time-activity curve from 48 h to infinity. Then it was integrated to infinity and the area under the curve was reported as the second part of the accumulated activity of each source organ. The time-activity curves after 48 h were considered mono-exponential functions due to this rational assumption that organs’ activities decrease with radioactive decay and biological elimination of the radionuclide in that organ (i.e. with the effective half-life of each organ). 

 Therefore, the exponent of any mono-exponential function represents the effective half-life of each organ. It should be noted that the effective half-lives of different organs were calculated as counting-based time-activity curves in this research.

 Finally, the effective dose was calculated according to the latest recommendations of the International Commission on Radiological Protection (ICRP) publication 103 (22), and was calculated from the following equation:

 E = $\sum_{T}w_{T}H(r_{T})$ (5)

 Where w_T_ is the weighting factor for tissue or organ T and HT, is the equivalent dose in tissue T, given in Sievert. Since the gamma rays and beta particles are involved in this research, the equivalent dose of tissues is directly calculated from the radiation absorbed dose of tissues (the radiation weighting factor of gamma and beta radiations is equal to 1). Decay correction was considered in all calculations such as percentage of the injected activities and time-activity curves.

## Results

^166^ ***Ho-HA's biodistribution in normal Wistar rats and humans***

 Holmium-166 radionuclide was obtained with a specific activity of 5.55–6.48 TBq/g. More than 80% of the labeled HA particles were in the desired size range of 20–60 µm. Radiolabeling yield increased to 99.2±0.2% when 5 mg/mL of HA was used. In vitro stability studies demonstrated satisfactory stability of the ^166^Ho-HA preparation in both normal saline and serum (~98% in normal saline after 7 days and more than 90% in human serum after 48 hours at 37 °C (10).

**Figure 1:**
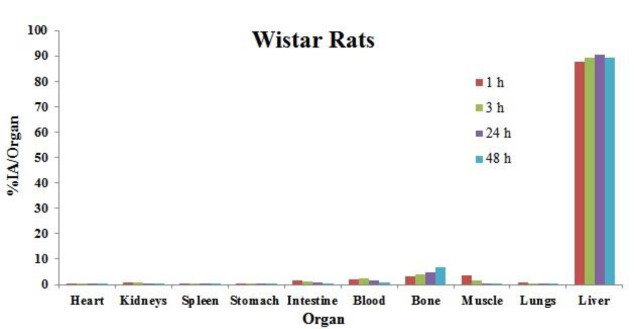
Percentage of the injected activity per organ (%IA/organ) of ^166^Ho-HA in normal Wistar rats

 The biodistribution of ^166^Ho-HA in different organs of Wistar rats up to 2 days (48 h) after injection is given in Figure 1. As shown in Figure 1, the major portion of injected activity through the hepatic artery remains in liver tissue (~ 90% of injected activity after 2 days) with insignificant uptake in any other major organs/tissues. It has an insignificant presence (<2% of injected activity) in blood circulation after 3 h and is rapidly taken up in bones after administration (up to 7% of injected activity). 

**Figure 2:**
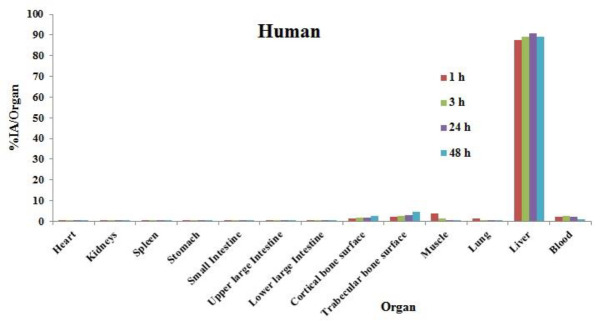
Percentage of injected activity per organ (%IA/organ) of ^166^Ho-HA in the adult man organs

 The percentage of injected activities per organ of human (%IA/organ) extrapolated from Wistar rats’ biodistribution data are given in Figure 2. As seen in Figure 2, most of the activity (more than %89) is retained in liver tissue. 

 In addition, more than 6% of the injected activity is accumulated on the surface of the trabecular (~4.46%) and cortical bones (~2.73%). The percent of injected activity in the remaining source organs is less than 0.5% after 48 hours post-injection.

**Figure 3:**
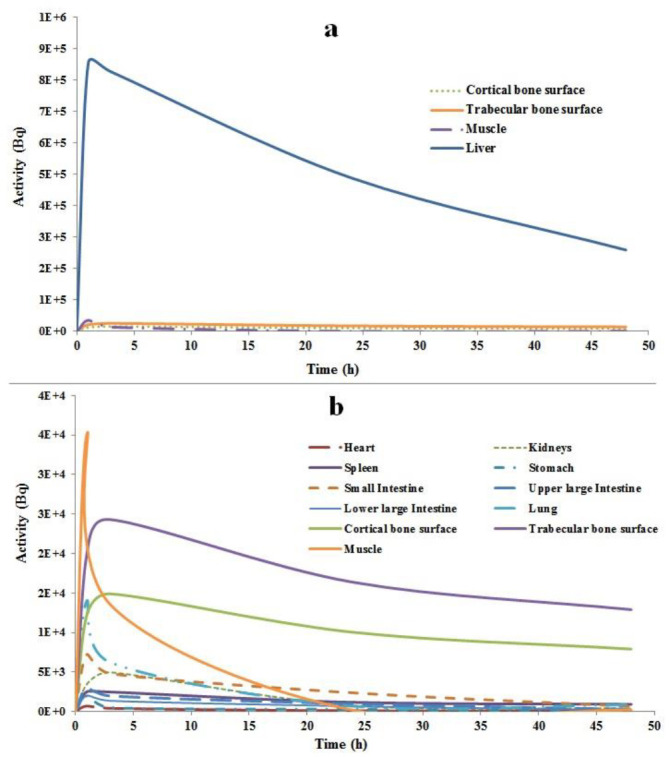
Time-activity curves of ^166^Ho-HA for source organs of the adult man. Liver, muscle, and cortical and trabecular bone surfaces in (**a**), and other source organs in (**b**)

 The time-activity curves for source organs of humans are given in Figure 3 a, and b, per injection of 1MBq of ^166^Ho-HA. Figure 3 a, shows that most of the activity is retained at liver tissue in each time interval and disintegrates with radiological half-life. Also, Figure 3 b, shows that the other tissues that include high amounts of holmium-166 activities are muscle, trabecular, and cortical bone surfaces, lung, and small intestine tissues respectively, although their values are not comparable with liver activity content. After one half-life of ^166^Ho radionuclide (26.8 h), there are insignificant activities in source organs except for liver and bone tissues.

***Radiation absorbed dose calculations***

 The accumulated activities (from injection time to infinity) in the source organs of the adult man per injection of 1 MBq of the ^166^Ho-HA radiopharmaceutical are given in Table 1. 

**Table 1:** The accumulated activities (MBq s) in the source organs of the adult man per injection of 1 MBq of ^166^Ho-HA

**Source organ**	**Accumulated activity**	**Effective half-life (h)**	**Source organ**	**Accumulated activity**	**Effective half-life (h)**
Heart	35	15.8	Lower large intestine (LLI) contents	143	14.4
Kidneys	336	12.8	Cortical bone surface	3870	49.5
Spleen	394	30.1	Trabecular bone surface	6315	49.5
Stomach contents	60	14.7	Muscle	780	6.2
Small intestine contents	504	14.4	Lungs	486	12.2
Upper large intestine (ULI) contents	208	14.4	Liver	125146	26.7

 Extracted effective half-lives of organs through fitting mono exponential equations on the activity-time curves are given in this table as well. R-squared values were more than 0.9 during the fitting process.

 As expected, the highest accumulated activity is observed in liver tissue, and activity on this tissue is approximately removed with an effective half-life equal to the radiological half-life.

 Because of the bone-avid features of the radiolabelled agent (hydroxyapatite), the trabecular and cortical bone surfaces would have the highest accumulated activities after liver tissue. Due to the large weight fraction of muscle tissue, the muscle-accumulated activity is the largest after the bone tissue.

 The estimated radiation absorbed dose of the selected target organs of the adult man along with the effective dose of ^166^Ho-HA radiopharmaceutical are presented in Table 2 per injection of 1 MBq activity. The effective dose can be considered as a rough estimate of the comparable whole-body irradiation.

**Table 2:** The radiation absorbed dose (mGy MBq^-1^) of the adult man target organs per injection of 1 MBq of ^166^Ho-HA radiopharmaceutical

**Target organs**	**Absorbed dose**	**Target organs**	**Absorbed dose**
Adrenal	0.013	Muscle	0.006
Brain	0.000	Pancreas	0.010
Breasts	0.002	Red marrow	0.179
Gallbladder wall	0.025	Osteogenic cells	0.383
Lower large intestine (LLI) wall	0.056	Skin	0.001
Small intestine	0.069	Spleen	0.239
Stomach wall	0.017	Testes	0.000
Upper large intestine (ULI) wall	0.055	Thymus	0.002
Heart wall	0.019	Thyroid	0.001
Kidneys	0.132	Urinary bladder wall	0.001
Liver	7.346	Total body	0.212
Lungs	0.060	Effective dose (mSv MBq^-1^)	0.390

 The highest radiation absorbed dose of ^166^Ho-HA radiopharmaceutical was delivered to liver tissue, as expected (7.346 mGy MBq^-1^). The results show that more than 84% of the absorbed dose is localized in liver tissue. 

 Skeletal tissue (Osteogenic cells and red bone marrow) and spleen were the next organs with high radiation absorbed dose. Red bone marrow and osteogenic cells radiation absorbed doses were estimated at 0.179 and 0.383 mGy MBq^-1^, respectively. In addition, the spleen received 0.239 mGy MBq^-1^ radiation absorbed dose. 

 Liver tissue received radiation absorbed dose of about 20 times the dose delivered to the osteogenic cells (the non-target organ with the highest radiation absorbed dose).

 The urinary system (kidney) is the next organ with the highest radiation absorbed dose in turn (about 0.132 mGy MBq^-1^), demonstrating rapid clearance of radiopharmaceuticals from the blood circulation. Also, the gastrointestinal tract receives considerable radiation absorbed dose compared with other remaining tissues which indicates the urinary and gastrointestinal systems as the main excretion routes of the ^166^Ho-HA radiopharmaceutical.

 The maximum tolerated doses (MTD) of liver, bone, bone marrow, and kidney are about 45, 70, 2, and 23 Gy, respectively (23-25). The activities corresponding to the maximum tolerated dose (MTD) by these tissues were given in Table 3 for ^166^Ho-HA and some bone-avid radiopharmaceuticals. Also, the maximum permissible administered activity into liver tissue for a 70 kg adult man was calculated for studied radiopharmaceuticals. The effective dose, as well as the radiation absorbed dose of some target and critical organs, were given in this table for comparison.

 As seen in Table 3, activity corresponding to MTD of the liver (target organ) for ^166^Ho-HA (6.13 GBq) is less than the activity required for MTD of bone, red bone marrow, and kidney tissues. While activity corresponding to MTD of bone (target organ) is more than activity required for MTD of bone marrow for all three bone pain palliation radiopharmaceuticals given in Table 3 which limits its high dose administration for therapeutic purposes.

 In addition, the results indicate that the injected activity of ^166^Ho-HA radiopharma-ceutical into the liver tissue should not exceed 87.5 MBq kg^-1^ (2.36 mCi kg^-1^) of body weight. 

 This administrated activity will result in a 45 Gy liver absorbed dose for a 70 kg adult man (inverses of 6.13 GBq activity administrated through intra-arterial radionuclide therapy). 

 The effective dose of ^166^Ho-HA radiopharma-ceutical (0.39 mSv/MBq) was obtained in the orders of bone avid beta-emitter radiopharma-ceuticals (less than 0.5 mSv/MBq).

**Table 3:** The MTDs and the maximum permissible administered activities of ^166^Ho-HA and some bone-avid radiopharmaceuticals

**Tissue**	^166^ **Ho-HA**	^153^ **Sm-EDTMP**	^166^ **Ho-EDTMP**	^177^ **Lu-EDTMP**
Activity (GBq) corresponding to MTD of the liver (45 Gy)	6.1	661.8	1500.0	2647.1
Activity (GBq) corresponding to MTD of bone (70 Gy)	182.9	17.3	22.7	11.3
Activity (GBq) corresponding to MTD of bone marrow (2 Gy)	11.2	1.4	1.4	1.9
Activity (GBq) corresponding to MTD of the kidney (23 Gy)	173.8	185.5	244.7	270.6
Max. administered activity for a 70 kg adult man (MBq kg^-1^)	87.5	20.3	20.1	27.1
Liver absorbed dose (mGy MBq^-1^)	7.346	0.068	0.030	0.017
Bone surface absorbed dose (mGy MBq^-1^)	0.383	4.037	3.085	6.162
Red marrow absorbed dose (mGy MBq^-1^)	0.179	1.413	1.435	1.067
Kidney absorbed dose (mGy MBq^-1^)	0.133	0.124	0.094	0.085
Effective dose (mSv MBq^-1^)	0.39	0.48	0.29	0.23
Reference	This work	(26)	(18,27)	(17)

## Discussion

^166^Ho-HA radiopharmaceutical showed massive retention in the liver tissue, which remained constant until the next 48 h. The remaining complex was rapidly cleaned of blood circulation (<3 h) and accumulated on bone tissue. No significant uptake was observed in other critical organs.

 Biocompatible mineral compounds such as hydroxyapatite, primarily tend to be localized uniformly on bone matrix if they enter the bloodstream after injection of a radiolabeled hydroxyapatite agent (10, 28, 29). 

 Hydroxyapatite Nano-crystals are uniformly distributed in the volume of both cancellous and compact bones. Because of the high sizes of hydroxyapatite (HA) particles in Das et al., (10) work (size range of 20–60 µm), most likely they will be deposited on the surface of both bone tissues. Following the recommendations of the ICRP, human bone surface uptake was considered as the trabecular (cancellous) and the cortical (compact) bones’ surface proportion at a ratio of 62% to 38% of the total skeletal surface (30). So in this research bone surface accumulation of ^166^Ho-HA radiopharma-ceutical was considered by this ratio.

 The biological elimination of ^166^Ho-HA radio-pharmaceutical from liver tissue can be ignored due to the strong adhesive property of particulates (HA) at liver tissue after intra-arterial administration of ^166^Ho-HA radio-pharmaceutical.

 Because of the high retention fraction of ^166^Ho-HA (about 98%) and its low excretion fraction (<2%) in the human body compared to bone pain palliation radiopharmaceuticals (retention fraction: <70%), the effective dose of this radiopharmaceutical (0.39 mSv MBq^-1^) is comparable with bone-avid radiopharma-ceuticals, even though the tissue weighting factor of the liver (0.04) is less than red bone marrow (0.12).

 Although the extrapolation between nonhuman primates (such as beagles, baboons, rabbits, mice, and rats) and human data may lead to overestimation or underestimation of absorbed dose, as shown in Table 2, the calculated radiation absorbed dose of tissues based on this method can be close to the real values, especially for target organs such as liver and other critical organs such as osteogenic cells, red marrow, and testes tissues. However, imaging studies are a principal method for the absorbed dose assessments of radiopharma-ceuticals in nuclear medicine.

## Conclusion

 Radiation absorbed dose, effective dose, and effective half-lives of holmium-166 labeled hydroxyapatite particulates were estimated in adult men based on biodistribution data in Wistar rats. ^166^Ho-HA radiopharmaceutical is intra-arterially administrated for the treatment of liver malignancies. The MIRD dose calculation method and the Sparks and Aydogan methodology were applied after activity-time curve extraction.

 The results indicate that the cancerous liver tissue will receive about 41 times more radiation dose than red bone marrow per unit of injected activity. The radiation absorbed dose of ^166^Ho-HA radiopharmaceutical for liver, red bone marrow, osteogenic cells, and spleen were estimated at 7.35, 0.18, 0.38, and 0.24 mGy MBq^-1^, respectively. The present study indicates that ^166^Ho-HA radiopharmaceutical would provide therapeutic efficacy for liver cancer and liver metastases with low undesired doses to other normal organs.
